# Cyclic on-chip bacteria separation and preconcentration

**DOI:** 10.1038/s41598-020-78298-y

**Published:** 2020-12-03

**Authors:** Vitaly V. Ryzhkov, Alexander V. Zverev, Vladimir V. Echeistov, Mikhail Andronic, Ilya A. Ryzhikov, Igor A. Budashov, Arkadiy V. Eremenko, Ilya N. Kurochkin, Ilya A. Rodionov

**Affiliations:** 1 FMN Laboratory, Bauman Moscow State Technical University, Moscow, Russia 105005; 2 Dukhov Research Institute of Automatics, Moscow, Russia 127055; 3 Institute for Theoretical and Applied Electromagnetics of Russian Academy of Sciences, Moscow, Russia 125412; 4 Faculty of Chemistry, M.V. Lomonosov Moscow State University, Moscow, Russia 119234; 5 N.M. Emanuel Institute of Biochemical Physics, Russian Academy of Sciences, Moscow, Russia 119334

**Keywords:** Lab-on-a-chip, Engineering

## Abstract

Nanoparticles and biological molecules high throughput robust separation is of significant interest in many healthcare and nanoscience industrial applications. In this work, we report an on-chip automatic efficient separation and preconcentration method of dissimilar sized particles within a microfluidic platform using integrated membrane valves controlled microfiltration. Micro-sized *E. coli* bacteria are sorted from nanoparticles and preconcentrated on a microfluidic chip with six integrated pneumatic valves (sub-100 nL dead volume) using hydrophilic PVDF filter with 0.45 μm pore diameter. The proposed on-chip automatic sorting sequence includes a sample filtration, dead volume washout and retentate backflush in reverse flow. We showed that pulse backflush mode and volume control can dramatically increase microparticles sorting and preconcentration efficiency. We demonstrate that at the optimal pulse backflush regime a separation efficiency of *E. coli* cells up to 81.33% at a separation throughput of 120.45 μL/min can be achieved. A trimmed mode when the backflush volume is twice smaller than the initial sample results in a preconcentration efficiency of *E. coli* cells up to 121.96% at a throughput of 80.93 μL/min. Finally, we propose a cyclic on-chip preconcentration method which demonstrates *E. coli* cells preconcentration efficiency of 536% at a throughput of 1.98 μL/min and 294% preconcentration efficiency at a 10.9 μL/min throughput.

## Introduction

The unique advantages of microfluidic analytical systems (MAS) over traditional laboratory methods are portability, operator-free operation, on-chip microcontrol capabilities, analysis time shortening and dramatic sample volumes shrinking^1^. Microfluidic systems which implement electrophoresis^2^, immunochromatography^3^, polymerase chain reaction^4^, droplet reactions^5^ and a number of other analysis methods have already demonstrated the superior efficiency to standard methods^6,7^. They have a strong potential to become the most powerful tools in mobile personalized healthcare, security and ecology applications in combination with state-of-art optical sensors^8–11^ based on extremely-high performance material platforms^12–14^.

However, almost all the optical sensing techniques (SERS, plasmonics, *etc.*) require careful sample preparation, dosing and protocol to ensure sensors’ performance. On the other hand, the functionality of many MAS can be significantly improved if pathogens present in the sample are separated, concentrated, and purified from substances that impede the accurate detection of pathogens and the determination of their concentrations at the sample preparation stage. The most obvious advantages of separated bacteria preconcentration are the possibility of separate detection of bacterial cells and other pathogenic nanoparticles, further reduction in sample volume, removal of possible inhibitors of the analytical reaction. In addition, it is possible to dramatically accelerate the analysis by reducing the duration or elimination of pre-cultivation stage^15,16^. Moreover, the isolation of pathogens from a sample in a “pure” form is of great interest for studying the genomics of pathogens and investigating the affinity for antibodies and peptides^17,18^.

Considering the foregoing, diagnostic polymerase chain reaction (PCR) is of particular interest due to the extreme power and speed of the method for diagnosis of microbial infections and genetic diseases, as well as for detecting microorganisms in environmental and biological samples. One of the hot spots for PCR applications is the detection of *E. coli* in human blood and plasma samples, as the incidence of extraintestinal *E. coli* (ExPEC) infections is increasing globally, which is a major public health concern^19^. The detection threshold for ExPEC using PCR in human plasma samples can be reduced by removing the two main inhibitors of PCR from the sample, i.e. immunoglobulin G (IgG) and lactoferrin^20^. Like the vast majority of microfluidic applications, the key separation parameters here are the separation efficiency (the ratio of the concentration that has been removed from the feed stream to the initial concentration in the sample) and the system throughput in μL/min^21^. Firstly, the higher separation efficiency provides more of the target bioparticle in the sample, which helps to carry out successful reactions with low analyte concentrations. Secondly, the higher separation throughput, less time the analysis takes. The sample volume for analysis in a microfluidic system usually lays between 15-1000 μL with typical value of 100 μL.

Modern microfluidic separation methods include various types of filtration^22^, particles size-dependent techniques based on microstructures capturing^23^, deterministic lateral in-flow displacement using objects with various shapes^24^, methods utilizing biomimetic effects^25^, affinity methods for analyte isolation^26^, inertia- and gravity-based techniques^27^, as well as on-chip magnetophoresis^28^, acoustophoresis^29^, electrophoresis^30^ and dielectrophoresis^31^.

In most microfluidic applications the key parameters of separation are separation efficiency (the ratio of concentration that has been removed from the feed stream to the initial concentration in the sample) and throughput of the system in μL/min. The prior challenge in the development of state-of-the-art microfluidic separation on-chip technique is to combine highest separation efficiency with best throughput^32^. One of the highest separation throughput of 400-1000 μL/min together with a high separation efficiency of 87-88% was reported in article^33^. The system intended for separation of 1 μm from 5-250 nm particles from lake water. However, no information about any particular bacteria or virus cultures separated using this set up was reported. In work^34^ an optofluidic nanoparticles sorting platform was used to separate 70 nm, 500 nm and 1 μm particles at the throughput of about 0.375 μL/min. One major drawback of this system is very low separation throughput that is inapplicable for routine usage in clinical practice. A passive separation system is demonstrated in article^35^. The system utilizes deterministic lateral displacement principle to separate 600 nm, 2 μm and 190 nm particles and achieves near 100% separation efficiency at the extremely low throughput of 0.01 μL/min. Separation system based on bacteria chemotaxis technique^36^ report 81% separation efficiency and around 0.013 μL/min throughput with 320 nm and 390 nm particles. Along with decent separation efficiency, the system operates too slowly for the majority of possible applications.

In this paper, a scalable microfluidic solution for batch micro- and nanoparticle separation based on microfiltration principle and robust on-chip valve flow control logic is proposed. Here we demonstrate a novel implementation of batch filtration method^37,38^ for on-chip automated separation and preconcentration of 1 mL colloid solution of *E. coli* bacteria cells. With the proposed method, we showed the separation efficiency of 81.33% at a throughput of 120.42 μL/min, which is absolutely competitive to the best published results. Furthermore, with the same microfluidic setup and suggested cyclic on-chip separation technique we achieved *E. coli* preconcentration efficiency of either 536% at a throughput of 1.98 μL/min or 294% at a throughput of 10.9 μL/min. The developed method allows micro- and nanoparticles separation from sub-mL liquid samples using automatic on-chip cyclic operations—at the end of each cycle, the microfluidic system gets back to the initial state and ready to separate the next sample portion. Due to such an approach, both filtrate (nanoparticles) and concentrate (bacteria) are available on the system’s output channels for subsequent processing or analyzing. Great scaling potential of on-chip platforms allows several microfluidic chips with purposefully chosen filters to be sequentially connected for two or more dissimilar sized particles separation and preconcentration.

## Experimental

### Microfluidic chip design

**Figure 1:**
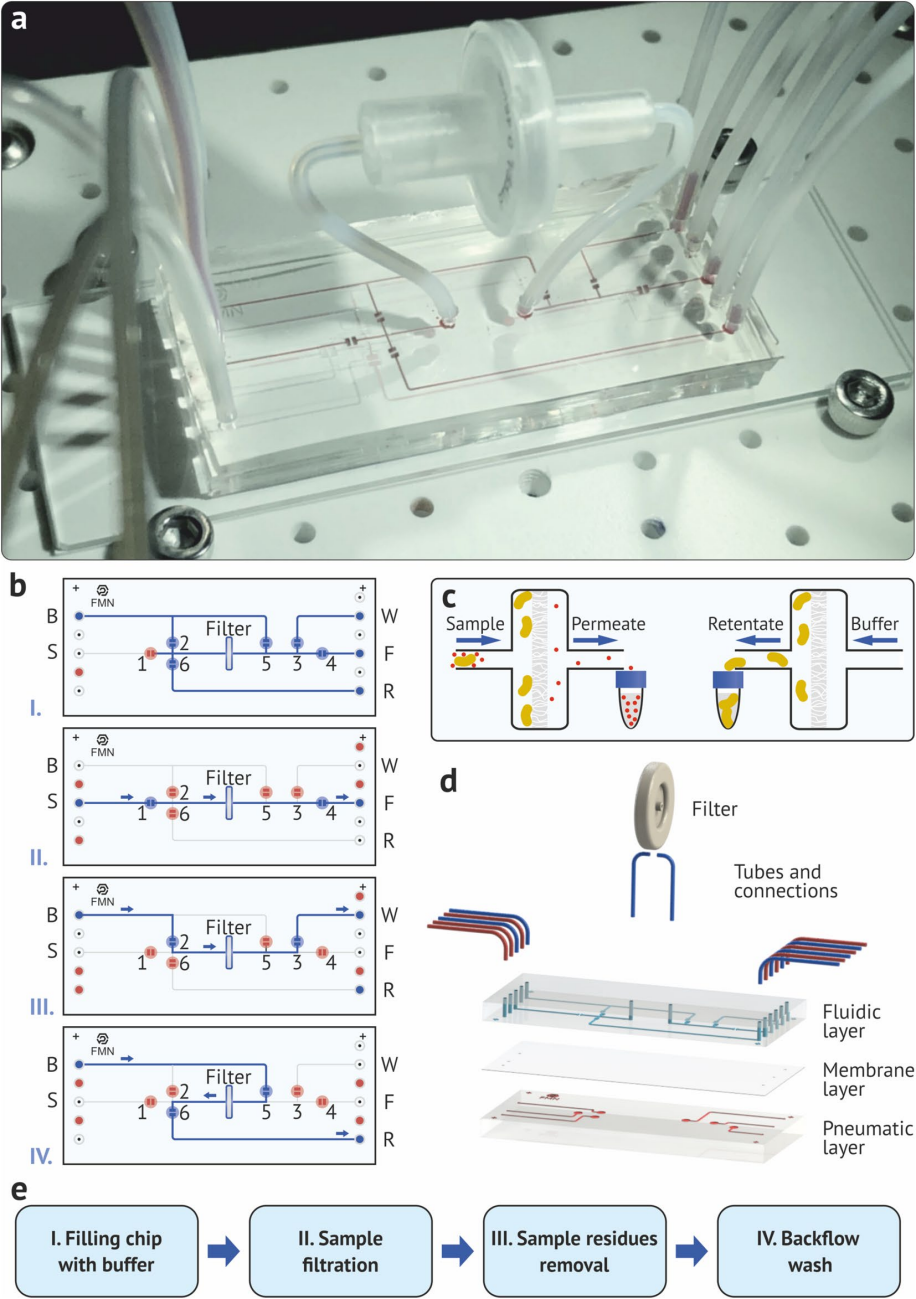
(**a**) Microfluidic separation chip with six valves on stage holder. (**b**) Valve opening/closing diagram at each step of the chip operation algorithm. (**c**) The principle of micro- and nanoparticles separation using filtration. (**d**) Microfluidic chip structure by layers. (**e**) The separation algorithm sequence.

The microfluidic chip for the separation of micro- and nanoparticles is shown in Fig. 1a. The chip design contains two input microchannels (marked blue of the left side of the chip) for supplying the initial sample (S) and buffer (B), six integrated membrane valves, a filter, six pneumatic valve control lines (all marked red) and three output microchannels for filtrate (F), concentrate (R), and buffer drain (W) (marked blue of the right side of the chip). The chip consists of three plasma bonded layers of PDMS (Fig. 1d) on a glass substrate. The lower pneumatic layer is intended for the topology of pneumatic microchannels for valve control; the middle layer is a 25 micrometer thick PDMS membrane, which is a movable working element of the valves, and the upper layer with the topology of microfluidic channels. For effective and fast separation of biological particles, the filter must provide a filtration rate of more than 200 μL/min at pressures that do not lead to the destruction of bacterial cells and do not increase their adhesion to the filter material. Considering the difference in the sizes of bacterial and viral cells and the experimentally established limiting pressures at which the bacterial cells do not rip, a round filter with a diameter of 13 mm and pores of 0.45 μm was chosen. The proposed method for on-chip separation includes the following sequence of operations (Fig. 1b):

- Valves 2-6 are open, valve 1 is closed, and microfluidic channels are filled with buffer.
- Valves 1 and 4 are open, the rest valves are closed. The sample is fed to the S (Sample) input, the sample is filtered, the filtrate with small particles (<100 nm) goes to the F (Filtrate) output, and particles larger than the pore size retain on the filter surface.
- Valves 2 and 3 are open, the rest valves are closed. The buffer is fed to input B (Buffer), flushes the channel and filter media from sample residues, the entire volume goes to output W (Waste).
- Valves 5 and 6 are open, the rest are closed. The predetermined volume of the buffer is fed to input B (Buffer); the bacterial cell concentrate is washed off the filter with reverse flow and fed to the output R (Retentate).

As a result, the small particles get to exit F (Filtrate), and the bacterial cells are backflushed to exit R (Retentate) with the required buffer volume. In this case, the concentration of «small» particles in the filtrate does not change, and the concentration of the bacterial cells in retentate could be increased, if the backflush volume was less than the sample.

### On-chip filtration and flow control

The basic principle of separation used in the microfluidic six-valve chip is shown in Fig. 1c, where the sample contains large particles with sizes of 0.5-2.0 μm (like bacteria cells), which are separated from small sub-100 nm particles (like viruses or proteins). At the first step, ”cake” type filtration occurs^39^—large particles are trapped on the filter, layering layer by layer on it, and small particles pass through the filter unhindered since the pore diameter of the filter is larger than their size. In the second step, the fine particle cleaning of the filter media with a buffer stream occurs. Finally, a reverse flow backflush is performed from the filter of the concentrate layers, in which large particles are in high concentration (Fig. 1e). To do this, the buffer is pumped in the reverse direction to detach the bigger particles from the filter.

**Figure 2:**
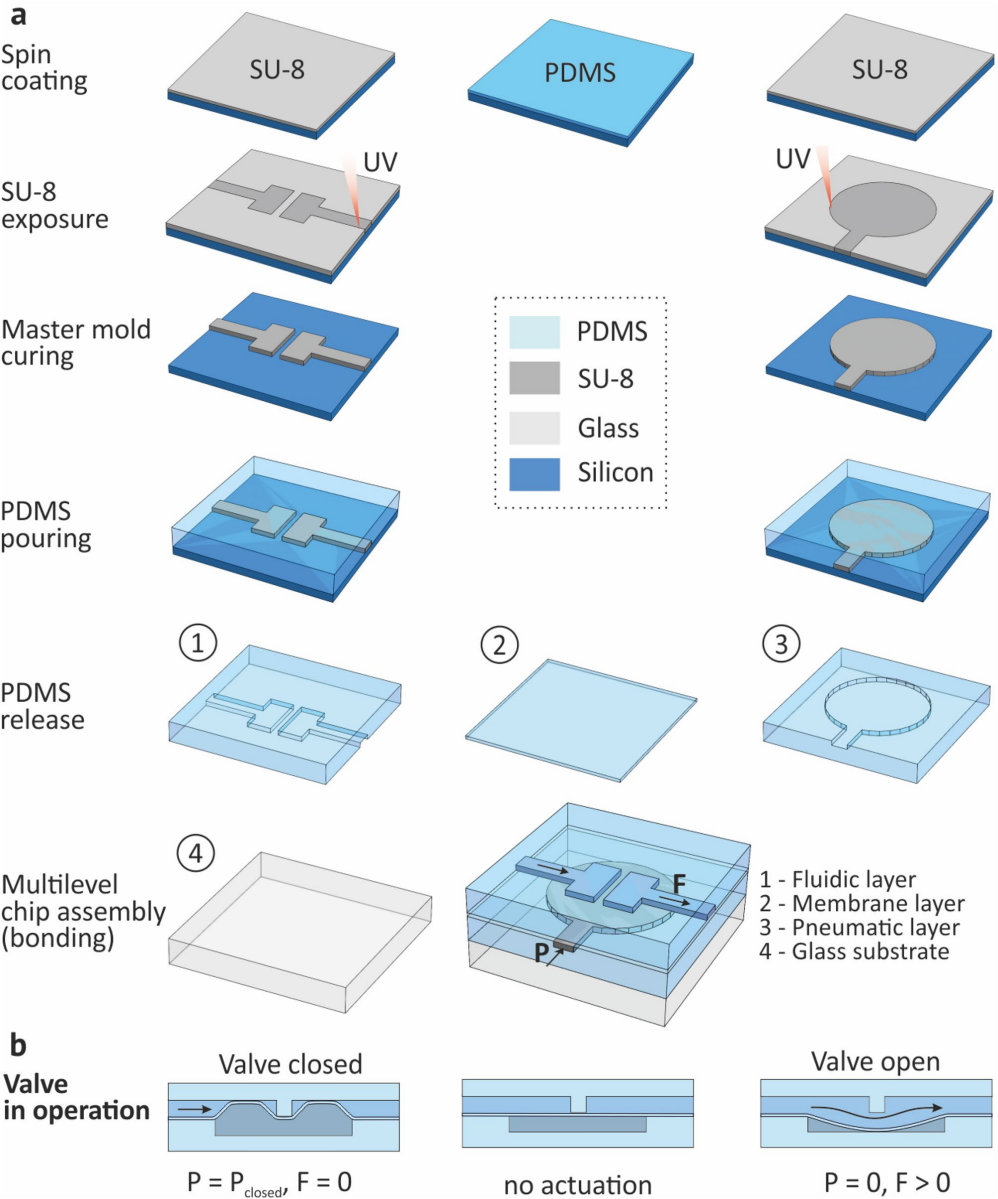
(**a**) Fabrication technology of multilayer microfluidic chip integrated with pneumatic valves. (**b**) Valve operation scheme.

On-chip liquids flow control is implemented using integrated pneumatic membrane valves. In contrast to the external valves, on-chip integrated pneumatic valves have sub-100 nL dead volume, which is critically important when operating with microliter volume samples. The operation principle of membrane valves is shown in Fig. 2b. Each valve consists of a microfluidic channel, separated by a membrane layer that blocks the fluid flow when excess pressure ($\hbox {P}> \hbox {P}_{close}$) is applied to the pneumatic line under the membrane. After pressure applied, the valve closes in approximately 100 μs stopping the fluid flow through the microfluidic channel. To move the valve back to the open state, the control pressure in the pneumatic line is reduced to zero, and the fluid flow in the microfluidic channel passes through the valve by bending the membrane.

The important characteristics for valves operation are control pressure and number of switching (open-close) cycles before valve degradation. In our earlier work^40^, we experimentally confirmed that our valves lifetime exceeds 10,000 switching cycles, which allows the automatic separation procedure to be performed more than 2,000 times on a single chip.

### Microfluidic chip fabrication

The microfluidic chip with six integrated pneumatic valves is fabricated using three layers of PDMS, which is bioinert and optically transparent (starting from a wavelength of 240 nm), which allows it to be used in combination with high performance optical biosensors. The fabrication technology consists of three main steps (Fig. 2a): litho-based casting of the microfluidic and pneumatic chip layers, valves membrane layer fabrication and sequential microassembly.

#### Fabrication of microfluidic and pneumatic chip layers

The microfluidic and pneumatic layers of the chip are made of PDMS by soft lithography using a mold made of SU-8 resist on a 100 mm silicon wafer (Siegert Wafer GmbH, Germany). The silicon wafer was cleaned in acetone, followed by washing in isopropyl alcohol and drying on a hot plate at a temperature of 120 $^{\circ }$C for 2 min. 5 mL of SU-8 3050 photoresist (Kayaku Advanced Materials, Inc., USA) was spin coated to the plate at 2800 rpm for 60 s, followed by drying on a plate at 95 $^{\circ }$C for 15 min. The thickness of the photoresist layer after the deposition process was 50 ± 2.5 μm. The resist was exposed by direct laser lithography (Heidelberg uPG101, Germany) with an exposure power of 50 mW (65% power, 20% filter) and a focus value of -5. Next, post-exposure bake was performed on a hot plate at a temperature of 65 $^{\circ }$C for 2 min, followed by heating to 95 $^{\circ }$C and holding the plate for 5 min. The development process was carried out using PGMEA (Kayaku Advanced Materials, Inc., USA) at a concentration of 100% for 7 min, followed by washing the plate in isopropyl alcohol. The photoresist hard bake process was carried out on a hot plate at a temperature of 175 $^{\circ }$C for 30 min.

PDMS base and hardener (Sylgard 184, Dow Corning, USA) were mixed in a mass ratio of 10:1. After thorough mixing, future PDMS was placed in a vacuum chamber for 10 min for degassing. Preliminarily silanized in Methyltrichlorosilane vapors (TCSM, Sigma-Aldrich, USA), a silicon wafer with SU-8 photoresist was placed in a petri dish and filled with PDMS mixture. The polymerization took place in a Model FD 23 drying oven (Binder GmbH, Germany) at a temperature of 100 $^{\circ }$C for 40 min. After detaching the PDMS print from the mold, PDMS was splitted into separate chips. Holes were made in each chip using a biopsy punch.

#### Fabrication of membrane layer

The membrane was fabricated by applying PDMS on a silicon wafer by spin coating followed by mechanical separation. Preliminary cleaning of the silicon wafer was carried out in acetone, followed by washing in isopropyl alcohol and drying on a hot plate at a temperature of 120 $^{\circ }$C for 2 min. The PDMS was applied at a rotation speed of 2000 rpm for 60 s, followed by drying on a hot plate at a temperature of 150 $^{\circ }$C for 10 min. The thickness of the membrane layer was 50 ± 2.5 μm. The membrane thickness is a critical parameter affecting membrane valve performance. During tests we found that the lower thickness does not provide enough mechanical durability to withstand high tensile stress for thousands of repetitions. Increase of the membrane thickness leads to higher actuation pressure and longer open/close time which affect negatively on the overall separation throughput. Moreover, thicker membranes with the same thickness uniformity are more difficult to fabricate. Therefore, the thickness of 50 μm is optimal for our application, as change in this parameter leads either to insufficient durability of the valves or to worse characteristics of separation.

#### Microfluidic chip assembly

Three prepared PDMS parts (microfluidic layer (FL), pneumatic layer (PL) and membrane (ML)) were placed in an oxygen plasma treatment chamber (Diener Pico, Germany) with a power of 11 W for 30 s. Then immediately after plasma treatment, the PDMS parts of the chip was assembled in a fiber-optic alignment system in order PL-ML-FL, starting from the bottom layer using alignment marks. The assembled chip was placed in a drying oven at a temperature of 100 $^{\circ }$C for 30 min to promote adhesion between the layers.

### Experimental setup

**Figure 3:**
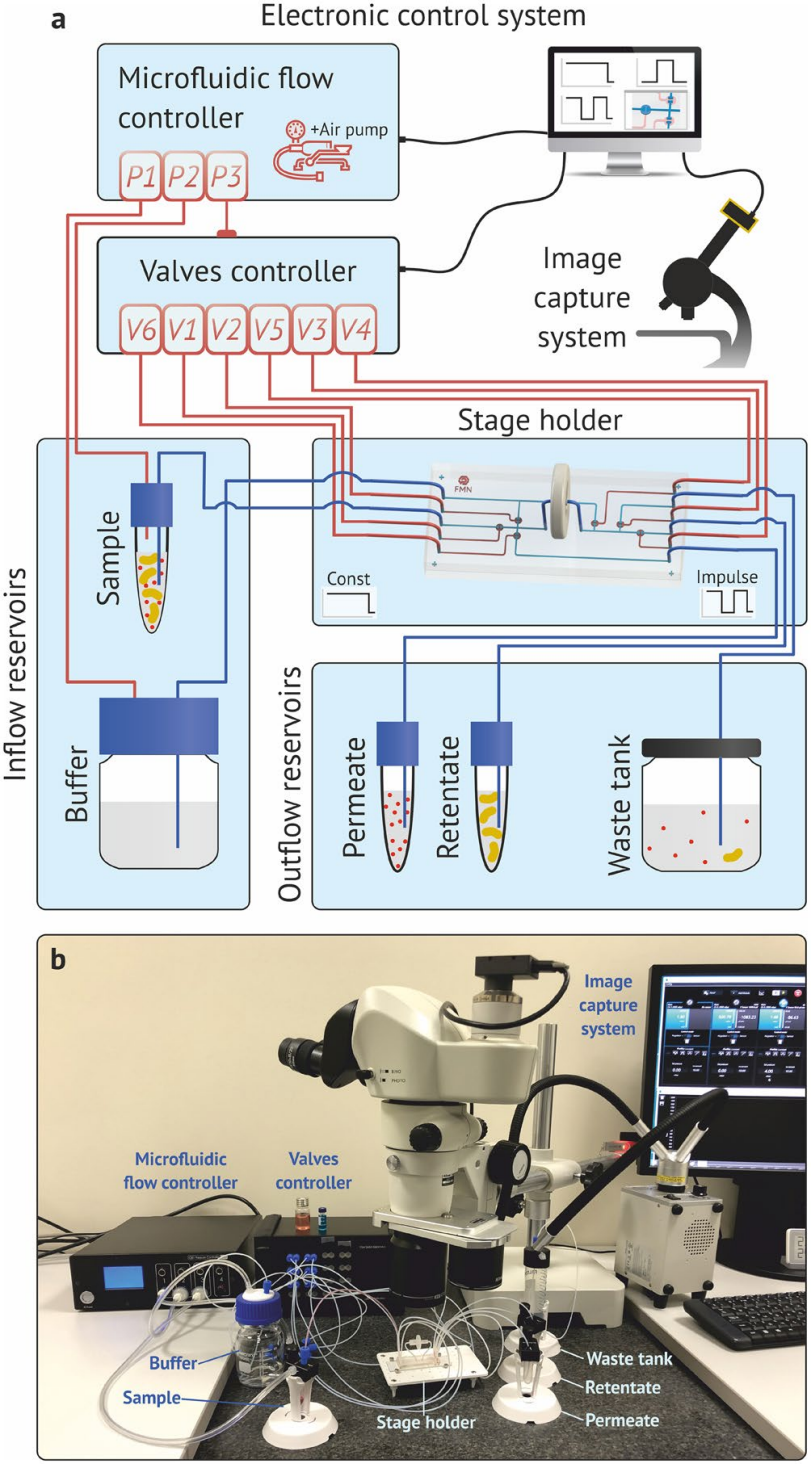
Scheme (**a**) and photography (**b**) of the experimental setup.

The experimental separation setup consisted of a control PC, a pneumatic microfluidic flow controller OB1 MK3 + pneumatic valves controller (Elveflow, France), an image capture system based on a high-speed Pixelink camera (Navitar, USA) and a Nikon SMZ800N microscope, the home-made stage holder, and reservoirs for inflow and outflow liquids. All stages of the experiment, including sample feed, control of the separation process, data acquisition and probe collecting after separation were carried out automatically (Fig. 3). The microfluidic flow controller was used to create overpressure in the sample (P2) and buffer (P1) reservoirs in order to supply a sample and buffer to a microfluidic chip, as well as the inlet pressure P3 supplying the pneumatic valves controller for integrated membrane valves actuation V1–V6. During the experiments, the valves actuation and the supply of liquids to the chip occurred according to a previously created algorithm in the developed home-made software package. The real-time valves control was carried out using the image capture system. All connections between the equipment units of the pneumatic and fluid parts, tanks, microfluidic chip and filter were made using Teflon (PTFE) tubes with an inner diameter of 0.5 mm.

At first we carried out a series of experiments with separation of 1 μm and 100 nm polystyrene beads (models for bacteria and virus/protein particles) in deionized water to demonstrate successful sorting using the proposed method. The purity of the two dissimilar sized fractions after separation was estimated using scanning electron microscopy (ZEISS MERLIN FE-SEM, Germany). In 10 independent experiments on processing 1 ml samples, the average purity for 1 μm and 100 nm beads were 91% and  100%, respectively. SEM images of the cryo-dried sample before and after separation can be found in Supplementary Figure B. After confirming the effectiveness of the method in the sorting of synthetic objects (polystyrene beads), we performed numerous experiments with *E. coli* cells to prove the concept with separation of living cells and determine the best operating regime for the proposed device.

Two materials of the syringe filter media were selected for the experiments: cellulose acetate (CA) and polyvinylidene fluoride (PVDF) (Acrodisc LC13 Syringe Filter, Pall Corporation, USA) with 13 mm effective filter diameter, 0.45 μm pore diameter and dead volume not exceeding 44 μL. After the first series of experiments, it was found that the use of CA filters is impractical because more than 70% of bacteria remain on the filter despite the reverse flow. We assume that the significant difference between the adhesion of bacteria to membranes made of PTFE and CA is explained by the higher surface energy of CA than PTFE, which causes a greater energy of interaction between the CA membrane and *E. coli* cells^41^. Therefore, all further experiments were carried out using hydrophilic PVDF filters.

Viral particles were excluded from the sample because the purpose of the experimental part was to demonstrate the effectiveness of the proposed separation method on a single chip, taking into account the possibility of scaling the system to two or more chips with smaller pore sizes in filters. We analyzed the possible effects of viruses or proteins presence in the sample, as well as degree of their influence on the results. We set up the experiment in such a way that the presence of viral particles did not affect the separation of bacteria significantly. Here are the main effects that viral particles may introduce in the workflow:

- virus/protein adhesion on PDMS,
- virus/protein adhesion on PVDF,
- virus/protein adhesion on bacteria,
- bacterial adhesion on PDMS,
- bacterial adhesion on PVDF,
- influence of virus/protein on bacteria size, shape, *etc.*).

The adsorption of bacteria on the chip materials (PDMS, PVDF) is extremely important parameter to minimize. Bacteria adhesion strongly depends on the roughness of the PDMS^42^ and on the preliminary processing of PVDF^43,44^. To minimize the influence of these effects on experiments with viral particles or proteins in the sample and without them, microfluidic chips with submicron roughness of the inner surface of the channels were made. Therefore, there is no significant adsorption of bioparticles on the PDMS. We also used a chemically treated PVDF filter to minimize bioparticle adhesion to the filter. In addition, due to the cyclical change in the direction of flow through the filter, the filtration efficiency remains nearly constant from cycle to cycle. Neutral acidity of the solution and low ion concentrations help to reduce the adsorption of bioparticles^45^, so we used deionized water ($\hbox {pH}=7\pm 0.15$) as a buffer in all experiments. Further, constant acidity above the isoelectric point of bacteria and viruses and the absence of ions in solution is important to ensure electrostatic repulsion and prevent bioparticles coagulation^46,47^.

The separated samples (Filtrate and Retentate) were collected in sterile DNA-clean strips and cuvettes used for PCR and photometry, the volume of the wash buffer was poured into a separate container. We used deionized water (DiW) prepared on the cleanroom water treatment plant (Hager + Elsässer, Germany) with the electrical resistance of about 18 MOhm·cm. The bacteria culture *Escherishia coli* DH5-Alpha was used in the experiments. The suspension based on an isotonic solution with a neutral pH with a bacterial concentration of 5·10^6^ cells/mL was sampled in cuvettes and used in experiments. Each experiment cycle $$($$sampling—experiment—measurements$$)$$ took no more than 16 hours.

### Filtration experiment

To investigate the efficiency of particle filtration using the developed microfluidic system, a series of experiments were carried out with the process parameters listed in Table 1. The microfluidic chip was connected with buffer (B), sample (S) and output (F, R, W) reservoirs using PTFE tubes. The pneumatic valve control system was connected using pneumatic tubes (V1-V6). Sample and buffer reservoirs were filled with 1 mL and 3 mL of bacteria suspension and DiW, respectively. Before each experiment, new 1.5 mL tubes were connected to the output channels F, R, W; during one of the series of experiments, the retentate was collected in separate cells of the microbial strip in a dropwise order. In each experiment at the first stage (Fig. 1b), all channels were filled with buffer under a pressure of 1000 mbar to remove bubbles from the chip. At the second stage, a sample with a given filtration pressure $\hbox {P}_F$ (Table 1) was supplied to the microfluidic chip from a tube with sample S. At a pressure of $\hbox {P}_F$ = 1000 mbar, the sample passed through the filter in less than 5 min. The filtrate was collected from output F in a separate container. At the third stage, the chip was washed with 200 μL of the buffer (which is > 15 internal volumes of the microfluidic chip) to remove the residues of the sample from the channels of the chip. At the fourth stage, bacteria were washed from the filter media with a given buffer volume (Table 1) by a reverse flow generated by constant pressure or pulse pressure in various modes (Table 1). After carrying out the entire sequence of separation operations, the concentration of bacteria in the retentate tube was measured. The concentration of bacteria was measured optically determining the absorption coefficient for the colloidal solution on a BioPhotometer D30 photometer (Eppendorf, Germany). The Semi-micro Original Eppendorf plastic cuvettes were used to measure concentrations in samples with a volume of more than 400 μL, and fiber-optic ultra-micro measuring cells TrayCell (Hellma, Germany) were used for experiments with retentate volumes of 10 μL. Calibration was performed using standard samples with known concentration for the optical paths of 10 mm and 1 mm, respectively. The calibration curve for the optical path of 10 mm is shown in Fig. 4c.

**Table 1:** *E. coli* separation experiment results.

#	Initial concentration C$_{in}$ (10^6^ cells/mL)	Filtration pressure P$_F$(mbar)	Backflush mode	Retentate concentration C$_R$ (10^6^ cells/mL)	Separation efficiency/preconcentration efficiency E$_S$ / E$_P$ (% of C$_{in}$)	Separation throughput T$_S$ (μL/min)
1	4.81	500	Constant.1 mL	1.615	33.59	46.96
2	5.81	900	Constant.2 mL	1.246	21.45	157.69
3	4.81	900	Constant.1 mL	2.324	48.34	113.26
4	5.81	900	Constant.1 mL	2.367	40.75	110.09
5	5.81	900	Constant.0.5 mL	4.741	81.61	70.19
6	4.81	1000	Constant.1 mL	1.831	38.09	125.10
7	5.25	1000	Constant.0.5 mL	6.397	$\hbox {E}_P$ 121.96	80.93
8	5.81	900	Pulse.2 mL	2.022	34.81	164.22
9	4.81	900	Pulse.1 mL	2.755	57.31	121.97
10	5.81	900	Pulse.1 mL	2.496	42.97	123.03
11	5.25	900	Pulse.1 mL	3.295	62.82	123.45
12	5.22	900	Pulse.1 mL	2.151	41.20	124.58
13	5.25	900	Pulse.1 mL	4.266	81.33	120.42
14	5.81	900	Pulse.0.5 mL	5.172	$\hbox {E}_P$ 89.03	81.36
15	5.25	1000	Pulse.1 mL	2.518	48.01	139.64
16	4.81	1000	Pulse.1 mL	2.906	60.45	140.80
17	5.25	1000	Pulse.1 mL	2.129	40.59	138.67
18	5.25	1000	Pulse.0.5 mL	6.054	$\hbox {E}_P$ 115.42	89.07

**Figure 4:**
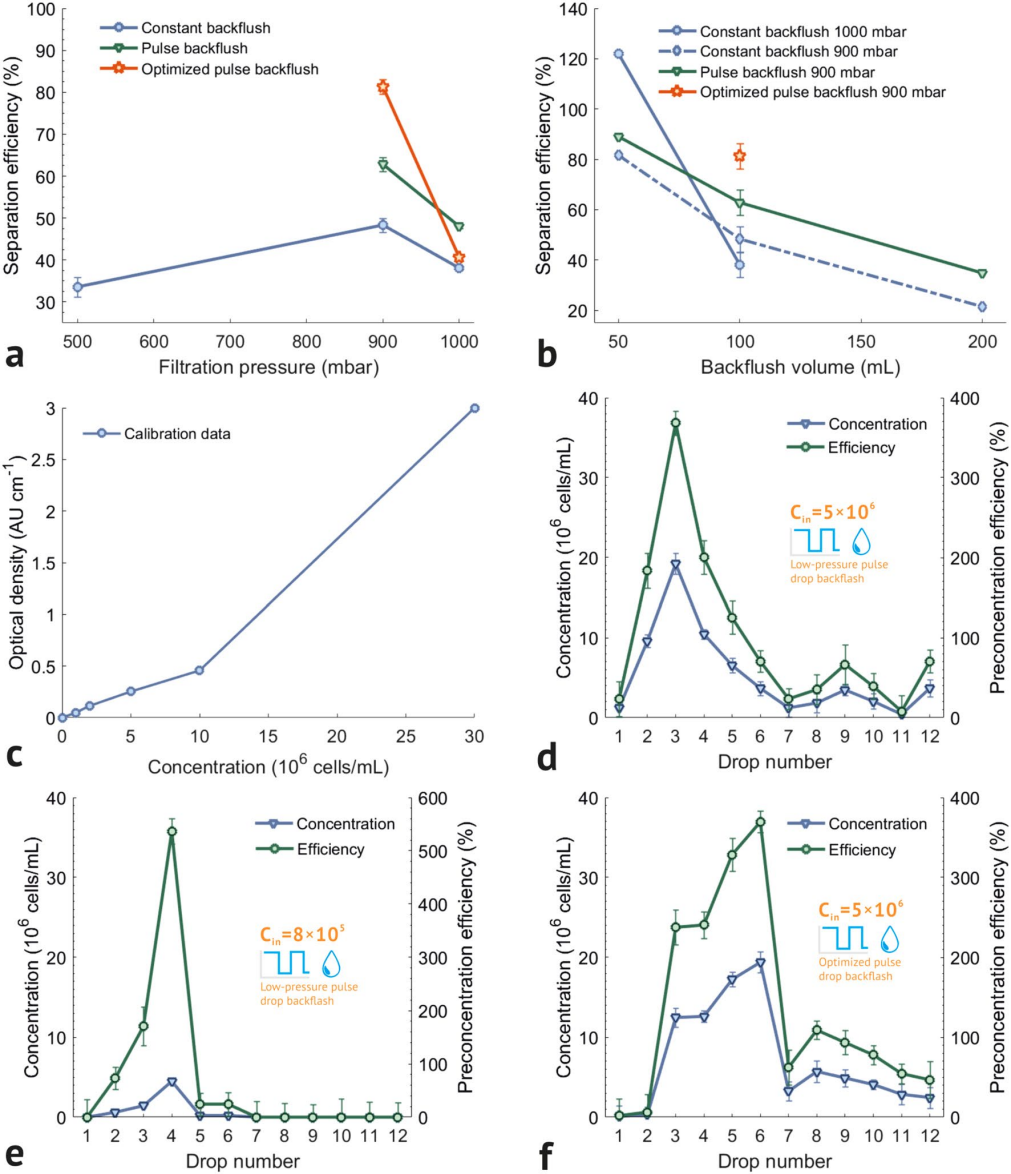
*E. coli* separation and preconcentration graphs. (**a**) Separation efficiency results with various filtration pressures. (**b**) Preconcentration efficiency with various backflush volume. (**c**) Calibration curve for 595 nm wavelength. Dropwise preconcentration of (**d**) 5·10^5^ cells/mL sample in low-pressure regime, (**e**) 8·10^5^ cells/mL sample in low-pressure regime, (**f**) 5·10^6^ cells/mL sample in optimized regime.

## Results and discussion

For separation efficiency ($\hbox {E}_S$) and preconcentration efficiency ($\hbox {E}_P$) calculations, the concentration of bacteria in retentate ($\hbox {C}_R$) was experimentally measured. Particle separation performance (separation throughput, $\hbox {T}_S$) directly depends on the filtration pressure ($\hbox {P}_F$). However, $\hbox {P}_F$ maximum value is limited by the durability of bacterial cell membranes and a decrease in the efficiency of particle separation during the backflush from the filter. The separation efficiency at different filtration pressures and constant reverse flow is shown in Fig. 4a (blue lines—constant backflush). In this experiment, the filtration of 1 mL of the sample was carried out with a $\hbox {P}_F$ pressure in the range from 500 to 1000 mbar, followed by backflush with 1 mL of buffer in constant mode with a $\hbox {P}_F$ pressure. There is a maximum sorting efficiency (60.3%) at $\hbox {P}_F$ = 900 mbar, which is associated with a low backflush efficiency at a pressure of 500 mbar and an increase in the coefficient of bacteria adhesion during filtration with a pressure of 1000 mbar. Similar dependencies are observed for all modes of constant and pulse backflush (green lines—pulse backflush and orange lines—optimized pulse backflush modes). The bacterial sorting performance was calculated as the ratio of the initial sample volume (1 mL) to the total time of the process (filtration, washing with buffer and backflush). With a constant reverse flow, sorting efficiency of 48.34% was achieved with $\hbox {T}_S$ of 113.26 μl/min.

To increase the number of bacteria successfully detached from the filter media a pulse backflush was suggested. In this case, the buffer is supplied with a pulse change in pressure with a period of 2 seconds: 1 second with filter pressure $\hbox {P}_F$ and 1 second with zero pressure. The use of the pulse backflush mode allows to increase the sorting efficiency by an average of more than 10% for all filtration pressures (Fig. 3a, 900 and 1000 mbar) and backflush volumes (Fig. 4b, 0.5, 1 and 2 μL).

To achieve higher separation efficiency and throughput, we tested sinusoidal, triangular and rectangular pulses with a duty cycle of 20% to 80% and a period of 2 s. We found that the best results were obtained using rectangular pulses with a duty cycle of 50% and a pressure between pulses equal to 70% of $\hbox {P}_F$ instead of zero pressure. Optimization of the shape of the supplied pulses and their period allows to achieve an additional 18.51% in $\hbox {E}_S$ (Fig. 4b, $\hbox {P}_F$ = 900 mbar): the sorting efficiency increase from 62.82 to 81.33% with $\hbox {T}_S$ of 120.42 μL/min. Filtration time in all the experiments was 300±30 s and backflush time was 200±30 s.

The detection limit of *E. coli* cells concentration in the first series of experiments was around 5·10^5^ cells/mL. Detection of lower bacterial concentrations is possible in a microcuvette (1.5–10 μL) of the photometer after preconcentration using centrifugation and resuspension methods. This approach is valid for a large volume of the initial sample. For the purpose of *E. coli* cells preconcentration, we carried out experiments with a variable volume of the backflush as well as cyclic sample separation in several cycles. The volume of the backflush is selected 50%, 100% and 200% of the initial volume (0.5 mL, 1 mL and 2 mL). When the backflush volume was 200%, a significant decrease in the concentration of bacteria occurred due to 2-fold dilution of the sample with buffer. Reducing the volume of the backflush down to 0.5 mL ($\hbox {P}_F$ = 900 mbar) allowed a significant increase in the concentration of bacteria for both constant (up to 81.61%, $\hbox {T}_S$ of 70.19 μL/min) and pulse (89.03%, $\hbox {T}_S$ of 81.36 μL/min) reverse flow modes.

At the filtration pressure of 1000 mbar and a backflush volume of 0.5 mL, we achieved bacteria cells preconcentration of 115.42%, $\hbox {T}_S$ of 89.07 μL/min for constant and 121.96%, $\hbox {T}_S$ of 80.93 μL/min for pulse backflush modes (Fig. 4b). The absence of any significant difference between concentrations in different backflush modes indicates that at a backflush pressure of 1000 mbar, all bacterial cells are washed off the filter with the first 0.5 mL of buffer.

To determine the optimal volume of backflush we carried out with dropwise collection of backflush in separate cells of sterile strips for microbiological analyzes. The volume of each microdrop was 10 μl, the filtration was performed at a pressure of 1000 mbar, reverse flushing in several regimes. The results of the experiment presented in Table A in Supplementary information section. Concentrations below detection limit of the photometer are indicated as “Low value”.

Figure 4d–f show the results of measuring the concentration of *E. coli* cells in each drop of backflush in two different regimes. Blue graphs represent concentrations measured in 10^6^ cells/mL and green match the same series of experiments and show preconcentration efficiency in % of initial concentration. The separation results of backflush with 0-600 mbar pressure pulses with 2 s period are shown in Fig. 4d. As we can see, in the third drop 4-fold preconcentration is achieved. Using from 2-nd to 5-th drops we can perform 2.19-fold preconcentration at throughput of 7.94 μL/min. Moreover, the same regime works with lower concentrations as well (Fig. 4e), which allows us 5.36-fold preconcentration of samples with bacteria concentrations near the lower detection limit of the photometer with throughput of 1.98 μL/min.

From these graphs, we could see space for further improvement of the process. The results of the first regime show a single drop with peak concentration, which gradually descends to minimal with a significantly less pronounced border.

To overcome this drawback more than 20 experiments with different backflush parameters were made. As a result, the optimized dropwise pulse backflush regime with 0-800 mbar pressure pulses was developed. The optimized backwash with a higher pressure pulses showed better results in localization of bacteria cells in reverse flow: we can see quite sharp borders between 2-3 and 6-7 drops where the majority of bacteria is presented. It means that the bulk of *E. coli* cells was separated from the filter media during the minimal amount of pulses, which is good, because it allows us to carry out 2.94-fold or 3.49-fold preconcentration with 10.9 or 5.45 μL/min throughput for 4-drop cutoff (3-6 drops) and 2-drop cutoff (5-6 drops).

The proposed method can be significantly improved by reducing the internal volume of the liquid system using a syringe filter with a smaller diameter with more accurate control and sampling. Furthermore, a thin filter membrane can be built into the chip^48^, which even further lower the internal volume of the microfluidic chip and introduce such highly useful effect as bubble removal. But at the same time this makes the entire chip disposable thus less cost-effective for real applications. The smaller droplet volume allows us to accurately collect 2-5 μL droplets carrying maximum concentration that can potentially be more than 10 times our current results which is more useful in real-world applications.

## Summary and conclusions

In this paper, we introduced a *cyclic batch sorting* method for on-chip automatic high throughput separation and preconcentration of dissimilar sized particles using microfiltration. Each cycle consisted of four main stages: bubble clots removal, microfiltration, removal of sample residues and washing in reverse flow. At the end of the cycle, our device was ready to sort the next batch of the sample. We operated with 1 mL portions of *E. coli* suspension in all experiments and carefully studied how such parameters as filtration pressure, backflush volume and pulse backflush regime affect the separation and preconcentration efficiency.

The microfluidic chip was fabricated using multilayer soft lithography technology of fully biocompatible materials and features with six integrated pressure actuated membrane valves with sub-100 nL dead volume.

We showed that reducing the volume and switching to pulse backflush pressure can significantly improve the efficiency of microparticle sorting and preconcentration. Hence, the proposed pulse backflush mode allowed us to raise the separation throughput without significantly increasing the filtration pressure, which is important for bacteria viability in applications linked to cell cultures can release factors that influence the proliferation and survival of neighbour cells. In addition, cell death can also affect tissue movement and morphogenesis by altering tissue tension in surrounding cells^49^. The rectangular pressure pulses have been found to be most effective in removing bacteria cells from the filter surface during the backflush. This parameter is crutial for PCR testing of samples with low analyte concentrations, as it increases the amount of the target organism in sample.

Using the proposed method we successfully separated 1 mL samples with *E. coli* bacteria with 81.33% efficiency at the throughput $\hbox {T}_S$ of 120.42 μL/min. The developed microfluidic chip is notable for its high versatility of applications due to its variable backflush volume. The efficiency of *E. coli* cells preconcentration up to 121.96% at $\hbox {T}_S$ of 80.93 μl/min was achieved by reducing the backflush volume by half. Preconcentration efficiency of 536% at the throughput of 1.98 μL/min and 294% at the 10.9 μL/min throughput for 8·10^5^–5·10^6^ cells/mL concentrations was achieved using proposed cyclic dropwise backflush mode.

The presented microfluidic platform performs a basic yet essential operation, which makes it highly application-flexible and scalable. Engineered and assembled with different microfluidic modules for sequent steps of sample preparation, chemical reactions and analyte detection, the results of this work will serve as a stepping-stone for the development of future cost-effective fully automated point-of-care devices.

## Supplementary information

Supplementary Information.
