# Supplementary material for: Cyclic on-chip bacteria separation and preconcentration
Source: Sci Rep. 2020 Dec 3;10:21107. doi: 10.1038/s41598-020-78298-y (PMC7713219; doi:10.1038/s41598-020-78298-y)
Supplement: Supplementary file 1 — Supplementary Information. [file 41598_2020_78298_MOESM1_ESM.docx]

**Cyclic on-chip bacteria separation and preconcentration**

**Vitaly V. Ryzhkov^1,+^, Alexander V. Zverev^1,2,+^, Vladimir V. Echeistov^1,2^, Mikhail Andronic^1^, Ilya A. Ryzhikov^1,3^, Igor A. Budashov^4^, Arkadiy V. Eremenko^5^, Ilya N. Kurochkin^4,5^ and Ilya A. Rodionov^1,2,^***

^1^ Bauman Moscow State Technical University, FMN Laboratory, Moscow, 105005, Russia

^2^ Dukhov Research Institute of Automatics, Moscow, 127055, Russia

^3^ Institute for Theoretical and Applied Electromagnetics of Russian Academy of Sciences, Moscow, 125412, Russia

^4^ M.V. Lomonosov Moscow State University, Faculty of Chemistry, Moscow, 119234, Russia

^5^ N.M. Emanuel Institute of Biochemical Physics, Russian Academy of Sciences, Moscow, 119334, Russia

* irodionov@bmstu.ru

+ these authors contributed equally to this work

|  | **Low-pressure dropwise pulse backflush C*_in_* = 5·10^6^ cells/mL** | | **Low-pressure dropwise pulse backflush C*_in_* = 8·10^5^ cells/mL** | | **Optimized pulse  drop backflush  C*_in_* = 5·10^6^ cells/mL** | |
| --- | --- | --- | --- | --- | --- | --- |
| **Drop #** | **C*_R_* (10^6^ cells/mL)** | **E*_P_* (% of C*_in_*)** | **C*_R_* (10^6^ cells/mL)** | **E*_P_* (% of C*_in_*)** | **C*_R_* (10^6^ cells/mL)** | **E*_P_* (% of C*_in_*)** |
| 1 | 1.224 | 23.45 | Low value | Low value | 0.102 | 1.94 |
| 2 | 9.592 | 183.73 | 0.612 | 73.14 | 0.327 | 6.23 |
| 3 | 19.231 | 368.37 | 1.429 | 170.79 | 12.462 | 237.59 |
| 4 | 10.462 | 200.4 | 4.49 | 536.63 | 12.615 | 240.51 |
| 5 | 6.531 | 125.1 | 0.204 | 24.38 | 17.231 | 328.52 |
| 6 | 3.673 | 70.36 | 0.204 | 24.38 | 19.385 | 369.58 |
| 7 | 1.224 | 23.45 | Low value | Low value | 3.265 | 62.25 |
| 8 | 1.837 | 35.19 | Low value | Low value | 5.714 | 108.94 |
| 9 | 3.469 | 66.45 | Low value | Low value | 4.898 | 93.38 |
| 10 | 2.041 | 39.1 | Low value | Low value | 4.082 | 77.83 |
| 11 | 0.408 | 7.82 | Low value | Low value | 2.857 | 54.47 |
| 12 | 3.673 | 70.36 | Low value | Low value | 2.449 | 46.69 |

**Supplementary Table A.** The results of *E.coli* dropwise preconcentration experiments.


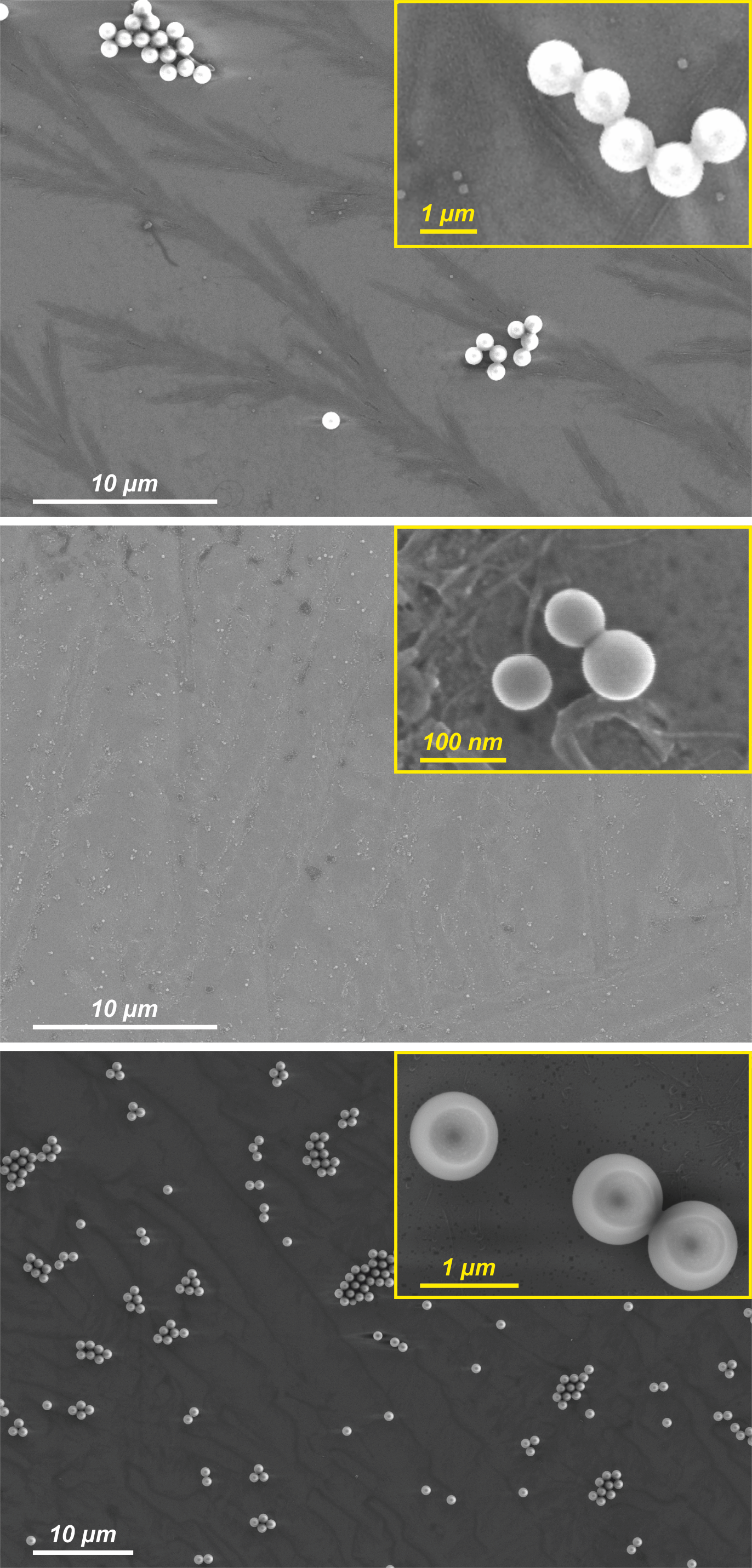


**Supplementary figure B.** SEM images of mixed sample with both 1 μm and 100 nm beads before separation (a) and permeate with 100 nm fraction (b), retentate with 1 μm fraction after separation.

**Supplementary Video C.** *E.coli* suspension pulsed flow in deionized water at zero pushing pressure between pulses after filtration.
